# Inadequate Immune Humoral Response against JC Virus in Progressive Multifocal Leukoencephalopathy Non-Survivors

**DOI:** 10.3390/v12121380

**Published:** 2020-12-02

**Authors:** Morgane Solis, Aurélien Guffroy, François Lersy, Eric Soulier, Floriane Gallais, Mathilde Renaud, Nawal Douiri, Xavier Argemi, Yves Hansmann, Jérôme De Sèze, Stéphane Kremer, Samira Fafi-Kremer

**Affiliations:** 1 Virology Laboratory, Strasbourg University Hospitals, 67000 Strasbourg, France; morgane.solis@chru-strasbourg.fr (M.S.); floriane.gallais@chru-strasbourg.fr (F.G.); 2 INSERM UMR-S 1109 LabEx TRANSPLANTEX, Strasbourg University, 67000 Strasbourg, France; soulier@unistra.fr; 3 Department of Clinical Immunology and Internal Medicine, National Reference Center for Systemic Autoimmune Diseases, Strasbourg University Hospitals, 67000 Strasbourg, France; aurelien.guffroy@chru-strasbourg.fr; 4 Service d’Imagerie 2, Strasbourg University Hospitals, 67000 Strasbourg, France; francois.lersy@hotmail.fr (F.L.); stephane.kremer@chru-strasbourg.fr (S.K.); 5 Neurology Department, Fédération de Médecine Translationnelle de Strasbourg (FMTS), Strasbourg University Hospitals, 67000 Strasbourg, France; m.renaud2@chru-nancy.fr (M.R.); jerome.deseze@chru-strasbourg.fr (J.D.S.); 6 Department of Infectious Diseases, Strasbourg University Hospitals, 67000 Strasbourg, France; n.douiri@clinique-rhena.fr (N.D.); xavier_argemi@hotmail.com (X.A.); yaves.hansmann@chru-strasbourg.fr (Y.H.); 7 Clinical Investigation Center, INSERM 1434, Strasbourg University Hospitals, 67000 Strasbourg, France; 8 Engineering Science, Computer Science and Imaging Laboratory (ICube), Integrative Multimodal Imaging in Healthcare, UMR 7357, University of Strasbourg-CNRS, 67000 Strasbourg, France

**Keywords:** immunology, viral infection, predictive marker

## Abstract

JC virus (JCV) causes progressive multifocal leukoencephalopathy (PML) in immunosuppressed patients. There is currently no effective specific antiviral treatment and PML management relies on immune restoration. Prognosis markers are crucially needed in this disease because of its high mortality rate. In this work, we investigated the compartmentalization of JCV strains as well as the humoral neutralizing response in various matrices to further understand the pathophysiology of PML and define markers of survival. Four patients were included, of which three died in the few months following PML onset. Cerebrospinal fluid (CSF) viral loads were the highest, with plasma samples having lower viral loads and urine samples being mostly negative. Whether at PML onset or during follow-up, neutralizing antibody (NAb) titers directed against the same autologous strain (genotype or mutant) were the highest in plasma, with CSF titers being on average 430-fold lower and urine titers 500-fold lower at the same timepoint. Plasma NAb titers against autologous genotype or mutant were lower in non-survivor patients, though no neutralization “blind spot” was observed. The surviving patient was followed up until nine months after PML onset and presented, at that time, an increase in neutralizing titers, from 38-fold against the autologous genotype to around 200-fold against PML mutants. Our results suggest that patients’ humoral neutralizing response against their autologous strain may play a role in PML outcome, with survivors developing high NAb titers in both plasma and CSF.

## 1. Introduction

JC virus (JCV) is the causative agent of the neurological disease progressive multifocal leukoencephalopathy (PML) [1]. JCV infects more than half of the population worldwide and persists in multiple body sites such as the urinary tract. However, PML develops primarily in at-risk primary or secondary immunosuppressed populations, such as patients seropositive for HIV, patients with primary immunodeficiencies (such as STAT1 GOF), patients with autoimmune diseases (such as sarcoidosis) or patients receiving immunomodulatory or immunosuppressive therapy [2,3,4,5,6]. One of the main drugs presenting PML as a major side effect is natalizumab, a drug used for multiple sclerosis and other autoimmune diseases. Other drugs, such as rituximab, alemtuzumab, fingolimod or mycophenolic acid (MPA), are more rarely associated with PML development.

PML diagnosis relies on clinical and imagery findings confirmed by the detection of the JCV genome in cerebrospinal fluid (CSF) by PCR [7]. JCV comprises eight genotypes; all genotypes can cause PML, even if genotypes 1 and 2B are sometimes more frequently reported in PML patients than in controls [8,9,10]. However, PML patients often present VP1 mutations such as L55F or S269F, which are specifically found in their CSF or blood and not in healthy individuals naturally excreting the virus in urine [11,12]. JCV serology using enzyme-linked-immunosorbant assay (ELISA) is not used as a diagnostic tool but rather as a prognostic marker of PML risk in patients receiving natalizumab [13,14]. However, as for other polyomaviruses, ELISA serology is not correlated with the neutralization capacity of the patient’s antibodies [15]. As there is currently no specific antiviral agent, PML treatment consists of immune response restoration by the discontinuation of immunomodulatory/immunosuppressive therapy or initiation of HAART in HIV+ patients, sometimes associated with IL-7 treatment or passive immunization [16]. Some recent observations also suggest restoring immunity against JCV with checkpoint inhibitor molecules against PD1 (pembrolizumab, nivolumab) [17,18]. This suggests that an insufficient anti-JCV immune response may be involved in PML development.

Indeed, recent work has shown that HIV+ PML patients display “blind spots” in their spectrum of neutralizing antibodies [19]: while they may present high neutralizing antibody (NAb) titers against several JCV genotypes, they have no or low NAb titers against JCV PML strains harboring mutations such as L55F or S269F, rendering them vulnerable to these specific strains. In addition, patients who do not develop neutralizing antibodies against their specific JCV strain after PML onset have a poor prognosis with high mortality. Our study aims to sequence JCV strains and to explore their compartmentalization in diverse PML contexts, as well as to analyze neutralizing antibody titers not only in plasma but also in other key matrices such as CSF, which may help define the prognosis markers of PML.

## 2. Materials and Methods 

### 2.1. Study Population

Four PML patients with longitudinal follow-up in various samples (CSF, plasma, urine) ranging from PML diagnosis to 2–9 months post-PML were included (7 to 12 samples/patient). Two of them had pre-PML samples available. In accordance with Jardé law n° 2012-300, this research did not require written consent.

### 2.2. JCV VP1 Sequencing

Sanger sequencing of the VP1 PCR product was performed after a nested PCR approach with the primers TGCTCCTCAATGGATGTTGCC (1395–1415) and GGTGCAGACACACAGGAAAAC (2689–2669) then CTTTTAGGGTTGTACGGGACTG (1420–1441) and AAAACCAAAGACCCCTCCCC (2630–2611).

### 2.3. Neutralization Assay

Anti-JCV NAb titers were measured at available timepoints before, during and after PML onset, in clinical samples such as plasma, CSF or urine. Neutralization assays were performed using a JCV pseudovirion system expressing the JCV capsid proteins as previously described for BKV [20,21,22]. The VP1 plasmid for JCV laboratory strain Mad1 was modified by site-directed mutagenesis to allow the production of JCV pseudovirions of different genotypes and strains (PML mutations L55F and S269F). The neutralization assay enables the quantification of antibody titers that functionally neutralize the infectivity of JCV pseudovirions. The neutralizing antibody titer was defined as the sample dilution that yielded 50% inhibition of pseudovirion infectivity (IC50) and was expressed as the log10 of the IC50. Samples were considered non-neutralizing if the 1:100 dilution (2.0 log10) for sera or 1:10 dilution (1.0 log10) for CSF and urine did not mediate at least a 50% luminometric signal reduction relative to the control condition without serum or with negative control (i.e., 50% neutralization of the reporter vector).

### 2.4. Statistical Analysis

The distributions of continuous data were compared using nonparametric Mann–Whitney and Kruskal–Wallis tests when comparing different groups of patients, and Wilcoxon signed-rank tests for paired comparisons. The distribution of categorical variables was compared using chi-square or Fisher’s exact tests. All analyses were performed using Graphpad software version 6.

## 3. Results

### 3.1. Patients and Viral Characteristics

Among the four patients included in this study, two had PML due to rituximab (P1, P2), one due to an association of rituximab with MPA (P4) and one due to HIV (P3) (Table 1). Genotyping showed JCV genotype 1B in three patients (P1, P2, P3) and genotype 3B in the last patient (P4). JCV VP1 sequencing showed that two patients had a JCV strain carrying the PML mutation S269F (P3, P4), and P1 presented a double population with a wildtype strain and another carrying the PML mutation L55F (Supplementary Figure S1), whereas P2 JCV strain did not display any mutation (though genotyping was only successful in urine samples). P2 was the only survivor.

### 3.2. JCV Strain Compartmentalization and Neutralizing Response

**Figure 1:**
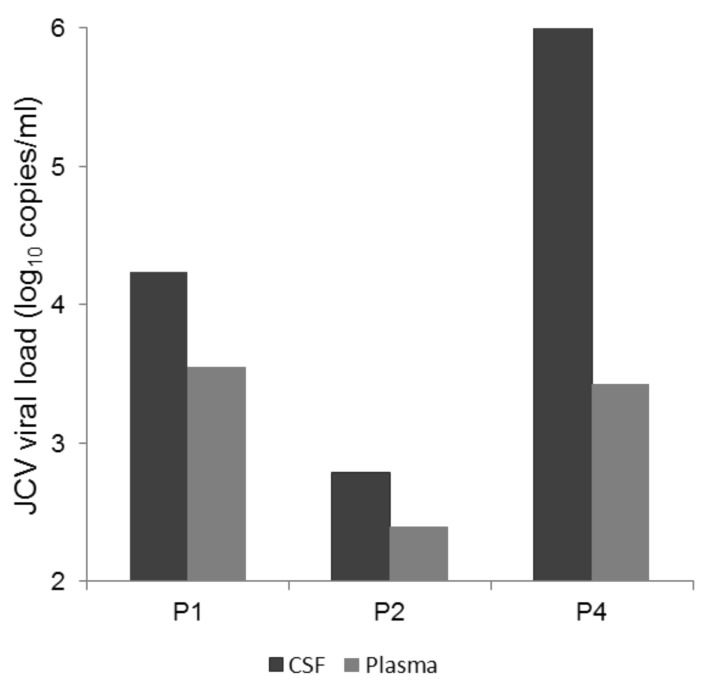
JC Virus (JCV) viral load in cerebrospinal fluid (CSF) and plasma at progressive multifocal leukoencephalopathy (PML) onset. Three patients (P1, P2 and P4) had both CSF and plasma samples taken at PML onset, which were all positive. P3 had a positive viral load in their CSF with no plasma sample taken for JCV viremia quantification. Mean viral load in CSF and plasma was 4.48 log_10_ copies/mL and 3.13 log_10_ copies/mL, respectively.

Both the CSF and plasma viral loads were positive at PML onset for P1, P2 and P4. P3 had a positive viral load in their CSF with no plasma sample taken for JCV viremia quantification. The CSF viral loads at PML onset ranged from 2.51 to 6.41 log_10_ copies/mL and were higher than the plasma viral load for all three patients (mean viral load in CSF and plasma = 4.48 log_10_ copies/mL and 3.13 log_10_ copies/mL, respectively) (Figure 1). The urine viral load was positive (4.11 log_10_ copies/mL) for only one out of three patients with urine available at PML onset.

Three patients underwent subsequent CSF and plasma testing in the following 1 to 2 months, which showed an increase in CSF viral load for all three (from +0.47 to +2.32 log_10_ copies/mL), whereas plasma viral loads remained stable or became negative. For three patients, VP1 sequences were available for both plasma and CSF and they were identical, with the same genotype and the same PML mutations found in both matrices (Table 1).

Whether at PML onset or during follow-up, NAb titers directed against the same autologous strain (genotype or mutant) were the highest in plasma, with CSF titers being on average 430-fold lower and urine titers 500-fold lower at the same timepoint. However, the differences could be even higher since some CSF and urine samples were negative, below the NAb detection threshold.

### 3.3. Lower NAb Titers in Non-Survivor PML Patients

**Figure 2:**
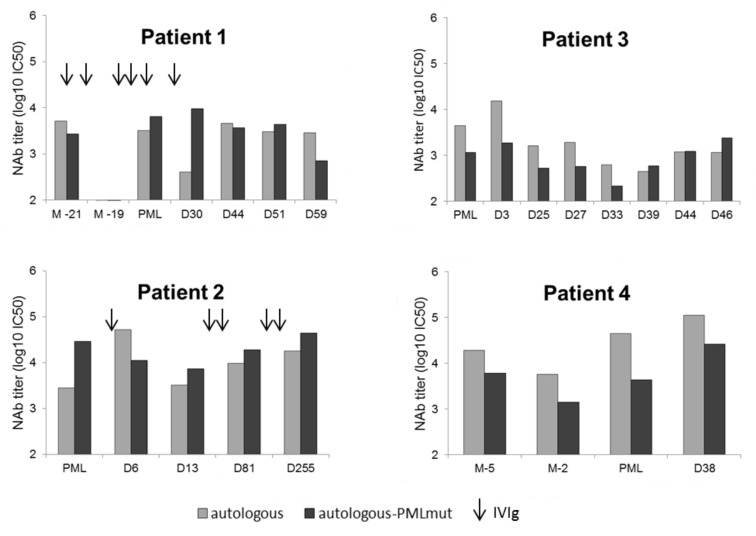
Evolution of neutralizing antibody (NAb) titers against autologous genotype and autologous mutant strains of survivor or non-survivor PML patients. NAb titers were measured in all plasma samples available for the four patients. P1 received intravenous immunoglobulins (IVIg) monthly, notably just before sampling at M-21, at M-20, a few days after sampling at M-19, at D-30, D0 and D30 after PML onset. P2 received IVIg at D6, D30, D77, D151 and D242 after PML onset.

Plasma NAb titers were compared during follow-up between survivor and non-survivor groups, both for antibodies against the autologous genotype and the autologous mutant (genotypic background + PML mutation) found in each patient (Figure 2). NAb titers directed against autologous genotype and autologous mutant were significantly lower in the non-survivor group (*p* = 0.015, Kruskall–Wallis test), especially against the autologous mutant (*p* = 0.0007, Mann–Whitney test) (log_10_ IC50 mean of 3.56 against the autologous genotype and 3.46 against the autologous mutant for the non-survivor group vs. 3.99 and 4.26 for the survivor, respectively).

### 3.4. Absence of “Blind Spots” in JCV Strains Neutralization Profiles at PML Onset

Despite NAb titers being lower in the non-survivor group during follow-up, all patients had detectable NAb titers against their autologous mutant at PML onset (Figure 2). Two patients presented lower titers against their mutant than their autologous genotype (3.06 vs. 3.65 log_10_ IC50 for P3 and 3.64 vs. 4.65 log_10_ IC50 for P4), while the two others presented higher titers against their mutant than their autologous genotype (3.81 vs. 3.51 log_10_ IC50 for P1 and 4.46 vs. 3.45 log_10_ IC50 for P2).

### 3.5. Intrathecal NAb Synthesis and PML Survival

**Figure 3:**
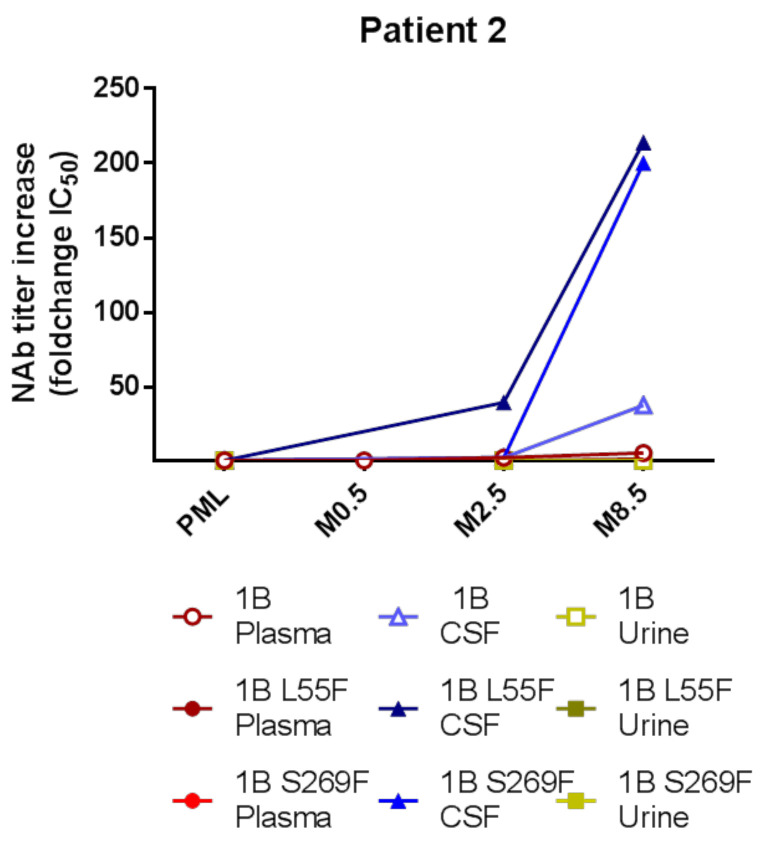
Kinetics of NAb titers against autologous genotype and mutant strains in different compartments in a PML survivor patient. Patient 2 developed PML and survived, with the last CSF sample being taken 8.5 months after PML onset. The autologous genotype for this patient was 1B.

For the survivor patient P2, samples up to 9 months post-PML including two CSF samples were available. Plasma and/or urine samples were also available at these timepoints. In plasma and urine, NAb titers against the autologous genotype or PML mutants L55F and S269F barely fluctuated during follow-up, whereas CSF NAb titers started to increase from 3- to 40-fold at 2.5 months and then even more drastically at 8.5 months (Figure 3), from a 38-fold increase against the autologous genotype to around 200-fold against PML mutants L55F and S269F.

## 4. Discussion

This is the first study exploring the compartmentalization of both viral strains and anti-JCV NAbs in order to understand JCV pathophysiology and define the prognosis markers of PML. Compartmentalization data showed that the CSF is the main location of the virus, with plasma samples having lower viral loads and urine samples being mostly negative. Indeed, about half of PML patients can have detectable JCV viral loads in plasma at PML onset [23], though viremia monitoring to predict PML development has been shown not to have any prognostic value [24,25] and is not used in routine practice. Sequence comparison between CSF and plasma showed that the same strain was present in both these compartments, as other studies have shown in natalizumab-treated PML patients [26]. Overall, NAb titers were higher in plasma but strongly increased during follow-up in the CSF of the survivor patient.

Despite lower NAb titers being observed during follow-up in non-survivor patients, no patient had a clear neutralization “blind spot” for their PML mutant at PML onset in this study. Conversely, a neutralization “blind spot” has been reported in HIV+ PML patients by Ray et al. [19] and has been suggested to be one of the factors driving PML development. These “blind spots” were defined as “little or no neutralization” (less than 2.5–3.0 log_10_ IC50) of the PML mutant compared to “robust neutralization” (3.0–4.0 log_10_ IC50 or more) of other strains. The authors suggested that these “blind spots” could make previously healthy patients more susceptible to PML after becoming immunosuppressed. However, it could be hypothesized that this “blind spot” may not be present in all PML contexts. While one of the patients included in this study developed PML due to HIV, the other three present several predisposing conditions for PML. All three were treated by rituximab about a year before PML onset. Rituximab is a class III PML agent, usually with no or very low potential risk of PML in the 2 years following administration, possibly due to its effect in blunting antibody responses. However, these patients had underlying clinical diagnoses with a predisposing risk of PML, such as granulomatous disease/common variable immunodeficiency (P1), Waldenström disease (P2) or immunosuppressive therapy after transplantation (P4). Our study shows that neutralization “blind spots” are not necessary for PML to develop, but they may reflect the state of the immune response against the PML mutant. Indeed, some of our patients received intravenous immunoglobulins (IVIg), which may have falsely elevated their NAb titers, masking “blind spots” but not the possible underlying deficiencies of other arms of the immune response against PML mutants predisposing to PML development.

Both P1 and P2 received IVIg for PML treatment. Interestingly, P1 also received IVIg infusions monthly for common variable immunodeficiency, notably with infusions just after M-19 sampling when no detectable neutralization was found, suggesting PML development consecutive to JCV primary infection. Only the survivor patient P2 reached NAb titers higher than 4 log_10_ IC50 in plasma, with titers being stable during follow-up, suggesting that IVIg cannot elevate NAbs at levels as high as these “natural” titers. However, while plasma and urine NAb titers stayed stable, the CSF NAb titers increased steadily for several months after PML for P2, correlating with imaging and clinical improvement, which is suggestive of intrathecal synthesis rather than blood–brain barrier crossing by antibodies. However, as only this one patient survived more than 2 months after PML, no conclusion can be drawn regarding the role of this intrathecal synthesis on clinical improvement and survival. In the previous study of Ray et al. [19], a 75-year old female patient with PML due to idiopathic CD4 lymphopenia was administered an experimental JCV vaccine and showed a roughly 100-fold increase in her plasma NAb titers against her autologous mutant virus, followed by JCV viremia decline and clinical improvement. Like our patient, the causal relationship between the improved neutralizing antibody response and the resolution of disease progression could not be ascertained.

One of the drawbacks of our study is that our cohort includes only four patients, though small cohorts are quite frequent for this disease. Patient follow-up was heterogenous due to the evolution of the disease, and samples could not be obtained at the same timepoints for all patients. Cellular immunity has historically been considered to be the main arm of defense against JCV-associated diseases due to the specific contexts of cellular immunosuppression in which PML develops. Studies have further shown that the impairment of the cellular response could be one of the main drivers of PML [27,28]. However, our results suggest that when the cellular response is impaired, the neutralizing response could come into play and have an important role in PML development and outcome. Furthermore, NAb measurement after PML onset could be a risk prediction parameter in addition to genetic susceptibility, with germline genetic risk variants having recently been identified [29]. In this work, our results help shed light on JCV compartmentalization during PML and suggest that there may be a better prognosis for patients who develop high NAb titers against their own strain, both in plasma and CSF. Still, further studies with more patients and a prospective design are actively warranted to confirm the pathophysiologic significance of the anti-JCV neutralizing response in PML. In this era of new immunosuppressive treatments and targeted therapies, in which the development of opportunistic diseases such as PML may increase, the search for easily identifiable PML prediction factors and prognostic markers is essential to improve the management of PML patients.

## Figures and Tables

**Table 1:** Patients’ characteristics.

Patient	PML Context	JCV Genotype	JCV Mutant	Survivor	IVIg Treatment
1	Granulomatous disease in common variable immunodeficiency treated by rituximab (last dose 14 months before PML onset)	1B	Double population wildtype/L55F (CSF/plasma)	no	yes
2	Waldenström disease treated by rituximab (last dose 10 months before PML onset)	1B	none detected (urine)	yes	yes
3	HIV	1B	S269F (CSF/plasma)	no	no
4	MPA for cardiac transplantation complicated by graft rejection treated by rituximab (last dose 9 months before PML onset)	3B	S269F (CSF/plasma)	no	no

## Supplementary Materials

The following are available online at https://www.mdpi.com/1999-4915/12/12/1380/s1, Figure S1: Double peaks at position L55F for P1.
