# Supplementary figures and images for: Inadequate Immune Humoral Response against JC Virus in Progressive Multifocal Leukoencephalopathy Non-Survivors
Source: Viruses. 2020 Dec 2;12(12):1380. doi: 10.3390/v12121380 (PMC7761562; doi:10.3390/v12121380)

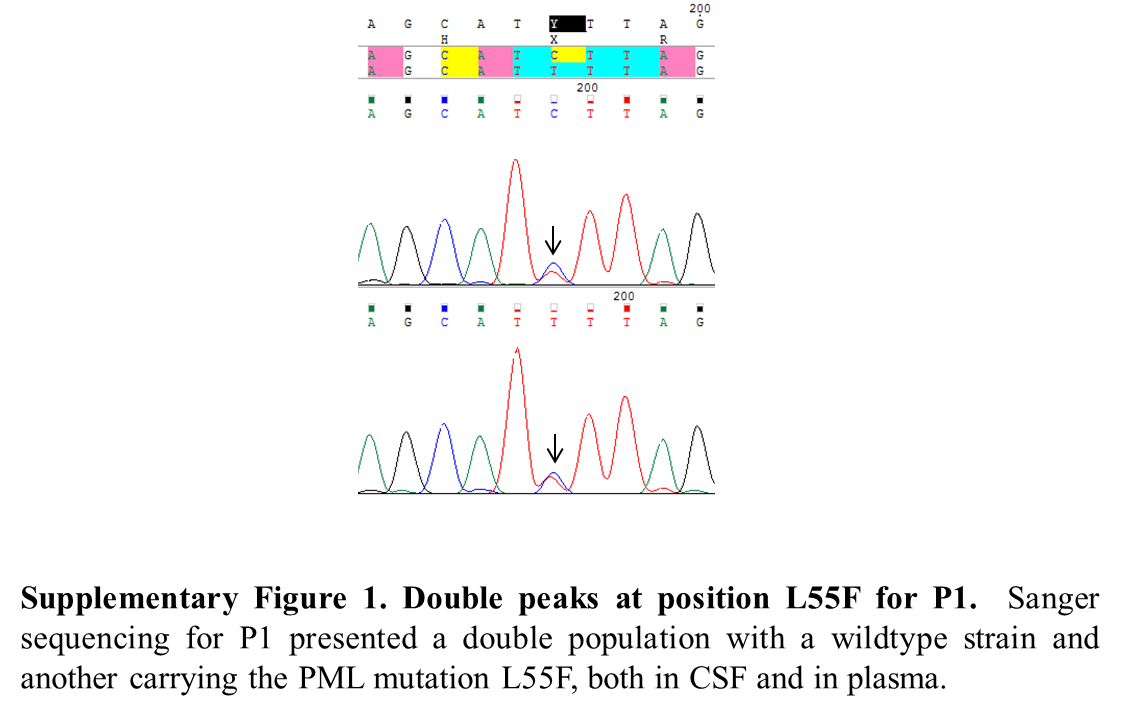

Supplement: Supplementary file 1 [file viruses-12-01380-s001.zip › viruses-982457-supplementary.tif]
